# Correlation between gene polymorphism in angiotensin II type 1 receptor and type 2 diabetes mellitus complicated by hypertension in a population of Inner Mongolia

**DOI:** 10.1186/s12881-020-01021-1

**Published:** 2020-04-19

**Authors:** Lina Hou, Xiaohong Quan, Xian Li, Xiulan Su

**Affiliations:** 1 Clinical Medical Research Center of the Affiliated Hospital, Inner Mongolia Medical University, Hohhot, 010050 Inner Mongolia, Autonomous Region People’s Republic of China; 2 Chifeng College of Medicine, Chifeng, Inner Mongolia, Autonomous Region People’s Republic of China

**Keywords:** Hypertension, Polymorphism, Type 2 diabetes mellitus, Angiotensin II type 1 receptor

## Abstract

**Background:**

The role of angiotensin II type 1 receptor (*AT1R*) as a key player in type 2 diabetes mellitus (T2DM) complicated with hypertension remains controversial. The present case-control study systematically investigated the association between gene the correct variation type in the angiotensin II type 1 receptor (*AT1R*) gene and type 2 diabetes mellitus complicated with hypertension in the Han population from the Inner Mongolia region, China.

**Method:**

Here, state which variants were analysis, including age, occupation, triglyceride, systolic, diastolic, sex, culture, marital status, smoking, alcohol, BMI (body mass index), SBP (systolic blood pressure), DBP (diastolic blood pressure), TG (triglyceride), TC (total cholesterol), HDL-C (high-density lipoprotein cholesterol), LDL-C (low-density lipoprotein cholesterol), FPG (fasting plasma glucose). Genomic DNA was extracted from samples from 202 type 2 diabetic patients with hypertension and 216 type 2 diabetic patients without hypertension.

**Results:**

Non-conditional regression analysis showed that in comparison with the TT genotype, the presence of the CC genotype for the T573 site of the AT1R gene increased the risk for diabetes mellitus complicated with hypertension by 3.219-fold (OR = 3.219, 95% CI: 1.042–9.941, *P* = 0.042). The results from multivariate linear regression analysis suggested the rs5182 polymorphism in the *AT1R* gene to be associated with diastolic blood pressure (*P* = 0.032). No other associations were found between the incidence of disease and the correct variation type at other sites of the *AT1R* gene.

**Conclusions:**

Our results suggest that the rs5182 polymorphism in the *AT1R* gene is associated with diabetes complicated by hypertension in the Han population of Inner Mongolia.

## Background

Diabetes mellitus and hypertension are often found in combination. The World Health Organization (WHO) reports that the prevalence of hypertension is 20 to 40% in diabetic patients [1]. Furthermore, research is China has found a prevalence of hypertension in diabetic patients as high as 49.9 to 60.6% [1]. The main cause of death in patients with diabetes and hypertension is the development of cardiovascular and cerebrovascular diseases.

Cardiovascular and cerebrovascular diseases are characterized by high morbidity, disability and mortality [2]. In China, there are approximately 290 million people suffering from cardiovascular and cerebrovascular diseases, at least 13 million stroke patients and at least 11 million coronary heart disease patients [3]. In fact, the number of deaths per year from cardiovascular and cerebrovascular diseases in China was higher than that caused by tumours and other morbid diseases and was at the top of the list of causes of death [4]. Therefore, the prevention and control of cardiovascular and cerebrovascular diseases have become a main priority. Some studies have shown that the incidence of cardiovascular and cerebrovascular disease in patients with hypertension complicated by diabetes is significantly higher than that in patients with simple diabetes [5, 6]. In addition, Ji et al. showed that the risk of developing cardiovascular and cerebrovascular diseases in patients with diabetes mellitus and hypertension is four times as high as that in healthy people [7]. Therefore, reducing the incidence of diabetes co-morbid with hypertension is likely to decrease the incidence of cardiovascular events. However, the pathogenesis of diabetes complicated with hypertension remains unclear.

The main components of the renin-angiotensin system (RAS) include angiotensinogen (AGT), renin (REN), angiotensin-converting enzyme (ACE), angiotensin, and angiotensin type 1 receptor (*AT1R*). The human *AT1R* gene is located on the long arm of chromosome 3 (3q21–25) and contains five exons and four introns. *AT1R* is mainly expressed in the vascular smooth muscle of the heart and other organs and tissues. *AT1R* mediates the contraction of blood vessels, stimulates the synthesis and release of aldosterone from the adrenal cortex, enhances water and sodium retention and increases blood pressure. In 1994, Bonnardeaux et al. confirmed the association between *AT1R* gene polymorphism and hypertension for the first time [8]. Subsequently, Stanković et al. found that a particular *AT1R* gene polymorphism was associated with hypertension in Serbian men [9]. In addition, Li et al. suggested the C allele of the *AT1R* gene to be closely associated with the occurrence of diabetes and diabetic nephropathy [10]. These findings suggest that *AT1R* may be involved in diabetes co-morbid with hypertension.

Diabetes co-morbid with hypertension is a complex chronic disease that is influenced by many factors, such as heredity and the environment. Lou et al. reported that the rs5186 polymorphism of the *AT1R* gene is associated with diabetes and hypertension in the Jiangsu Han population and that there was a correlation of the C allele with type 2 diabetes with an increased risk of hypertension [11]. In contrast, Lesage et al. did not find an association between *AT1R* gene the correct variation type and diabetes mellitus complicated by hypertension in French Caucasians [12]. As research about associations between angiotensin type 1 receptor (*AGT1R*) and angiotensinogen (*AGT*) genotypes in type 2 diabetes, the resluts shows that no significant associations were seen between AGT1R T573 C-allele and renal dysfunction or coronary heart disease (CHD) [13]. The *AT1R-A1166C* polymorphisms examined in the RAS may modulate the risk factors associated with cardiovascular-renal disease [14]. The angiotensin type 1 receptor (*AT1R A1166C*) genotyping of renin-angiotensin system genes could become an important part of the clinical process of risk identification for T2DM in Tunisian population [15]. As the above studies are inconsistent, the possible association between *AT1R* gene the correct variation type and diabetes mellitus complicated by hypertension remains unclear. A possible reason for these inconsistent results might be that each study focused on regions of the world with distinct environments and different genetic backgrounds.

The present study investigates a possible association between *AT1R* gene the correct variation type and the pathogenesis of diabetes mellitus complicated by hypertension in the Han population of Inner Mongolia. In this study, we compared *AT1R* the correct variation type present in patients with diabetes mellitus (type 2) with hypertension to those of patients with diabetes without hypertension (control group). The aim of this study was to contribute to the development of a diagnostic tool for type 2 diabetes complicated with hypertension that might be used in the prevention and control of cardiovascular and cerebrovascular diseases.

## Methods

### Sampling of study participants

For this study, a sample of Han patients was recruited in the outpatient clinic of Inner Mongolia Medical University Affiliated Hospital from October 2016 to September 2017. The consent obtained was verbal and approved by the Medical Ethics Committee of Inner Mongolia Medical University (number:YKD2012018). A total of 202 type 2 diabetic patients with hypertension (104 males and 98 females) and 216 type 2 diabetic patients without hypertension (128 males and 88 females) were selected with ages ranging from 50 to 70 years, shown in the Supplementary file 1. The selected population covered patients belonging to three generations with no tribal intermarriage history and who were born in the Inner Mongolia Autonomous Region, China. Diabetes was diagnosed according to 1999 World Health Organization Guidelines [16], and hypertension was diagnosed according to 2010 Chinese Guidelines for the management of hypertension [17].

The inclusion criteria for the case group were as follows: (1) patients diagnosed with type 2 diabetes complicated with hypertension; (2) patients who agreed to participate in the study and signed an informed consent form. The exclusion criteria for the case group were as follows: (1) patients who refused to sign the informed consent form; (2) patients with macrovascular disease, primary aldosteronism, renal disease, pheochromocytoma and other high blood pressure-underlying reasons.

The inclusion criteria for the control group were as follows: (1) patients diagnosed with type 2 diabetes without hypertension; (2) patients who agreed to participate in the study and signed the informed consent form. The exclusion criteria for the control group: (1) patients who refused to sign the informed consent form; (2) patients with cancer, liver and kidney disease and thyroid disease.

The interview lead questionnaires were conducted. The content of the questionnaire included the following: (1) demographic characteristics such as sex, age, education level (primary, junior high school, and high school education), marital status (married, living together, single, divorced, separated, widowed), and occupation (farming, factory, office workers); (2) lifestyle choices such as smoking (no or yes: ≥ 1 cigarette/day for at least 1 year), and drinking (no, yes: ≥ 50 g or more alcohol/day for at least 1 year); (3) past and family medical history (diabetes, high blood pressure, hyperlipidaemia, stroke, coronary heart disease, and kidney disease, among others).

Physical examination was carried out by medical staff specialized in physical examination. Measurements included height, weight, and waist and hip circumferences. Body mass index (BMI) was calculated using the formula BMI = body weight (kg) / height squared (m^2^), according to the “Guide to Prevention and Control of Overweight and Obesity in Adults in China” [18]. The BMI classes considered were 18.5 ≤ BMI < 24 kg / m^2^ for normal weight, 24 ≤ BMI < 28 kg / m^2^ for overweight, ≥ 28 kg / m^2^ for obesity.

Blood samples (10 ml) were obtained from the antecubital vein after ≥8 h of fasting of all participants, including 202 type 2 diabetic patients with hypertension (104 males and 98 females) and 216 type 2 diabetic patients without hypertension (128 males and 88 females). Each blood sample was collected into two PE tubes, which were then centrifuged for 10 min (3000 rpm). One sample (tube) was used for the determination of biochemical indicators, and the other was stored at − 80 °C for later reference. Blood biochemical indicators were quantified in the laboratory department of the Inner Mongolia Medical University Hospital and included plasma total cholesterol (TC), triglyceride (TG), high-density lipoprotein cholesterol (HDL-C), and low-density lipoprotein cholesterol (LDL-C), among others, which were measured using a Roche c8000 automatic biochemical analyzer (Roche, USA).

### DNA extraction and tagSNP genotyping

We focused on 3 tagSNPs of the *AT1R* gene (rs5182, rs5186, rs35533650) for genotyping purposes. These SNPs were selected based on a pairwise r^2^ ≥ 0.5 value and allele frequency (MAF) ≥ 0.05 according to the Chinese HapMap database (http://www.hapmap.org) [19].

Genomic DNA was extracted from peripheral blood leukocytes using a commercial blood DNA extraction kit (Genomic DNA purification kit; Aidlab Biotechnologies Co., Ltd., Beijing, China) and stored at − 20 °C. Genotyping of the three polymorphic sites (rs5182, rs5186 and rs35533650) was based on imLDR technology (Genesky Biotechnologies Inc., Shanghai, China). Multiplex PCR reactions were performed using multiplex PCR to amplify regions within the target SNP site. The sequences of the PCR primers used in the current study are listed in Table 1. Subsequently, the amplified product was purified using shrimp alkaline enzymes (Exo I / SAP) and an exonuclease to obtain a DNA template for subsequent ligase reactions. The ligation products were separated by capillary electrophoresis using an ABI 3730XL instrument and analysed with GeneMapper 4.1 software.

**Table 1:** PCR primers used in the current study

TagSNPs	Forward(5′- 3′)	Reverse(5′- 3′)
rs5182	TTGGCCAGTTTGCCAGCTATAA	GCCTTCTTTAGGGCCTTCCAAAT
rs5186	CCAAAGCCAAATCCCACTCAA	GCTTTAGAAAAGTCGGTTCAGTCCA
rs35533650	GCTGCACTGGTCCCAAGTAGT	CTTTGCCAGATTTTAATCAATTAACAGC

### Statistical analysis

Quantitative data were described as the mean ± standard deviation. Qualitative data were described by the adoption rate or composition ratio. The independent sample t-test, χ2 test and rank sum test were used to compare demographic characteristics, physical examination results and biochemical detection indexes. Using the Hardy-Weinberg Principle, expected genotypic frequencies were calculated and compared with observed genotypic frequencies. The goodness-of-fit test was employed to determine Hardy-Weinberg equilibrium (HWE) for the sample population. Allele frequencies were calculated based on genotypic frequencies. The χ2 test was used to compare genotypic and allele frequencies between the case group and control group. Non-conditional logistic regression analysis was employed to calculate the odds ratio (OR) with a 95% confidence interval (CI) for each tested *AT1R* gene polymorphism. Multivariate linear regression analysis between each tested AT1R gene polymorphism and systolic and diastolic blood pressure was undertaken. Statistical analysis was conducted using SPSS19.0 statistical software (SPSS Inc., Chicago, IL, USA).

## Results

### Demographic characteristics of the study participants

As shown in Table 2, the age and occupational differences between the case and the control groups were statistically significant (*P* < 0.05). There were no significant differences in sex, educational level, marital status, smoking status, or drinking status between the two groups (*P* > 0.05).

**Table 2:** Demographic characteristics of the study participants

Variables	Case	Control	t/χ^2^	*P*
	(*n* = 202)	(*n* = 216)		
Sex, n(%)			2.554	0.110
Male	104(51.49)	128(59.26)		
Female	98(48.51)	88(40.74)		
Age(years)	58.54 ± 9.76	53.04 ± 11.86	−3.983	<0.001*
Degree of education, n(%)			5.821	0.054
Primary school and below	44(21.78)	62(28.70)		
Junior high school	37(18.32)	50(23.15)		
High school and above	121(59.90)	104(48.15)		
Marital status, n(%)			1.272	0.259
Married, living together	184(91.09)	203(93.98)		
Unmarried, Widowed Divorced, Separated	18(8.91)	13(6.02)		
Occupation, n(%)			8.331	0.016*
Worker	63(31.19)	41(18.98)		
Farmer	22(10.89)	27(12.50)		
Office staff	117(57.92)	148(68.52)		
Smoking, n (%)	73(36.14)	86(39.81)	0.599	0.439
Alcohol consumption, n (%)	66(32.67)	67(31.02)	0.132	0.717

Significance: * = *P* < 0.05

### Physical examination and biochemical analysis of the participants

As shown in Table 3, all physical examination indexes for the case group, including BMI, SBP and DBP, were significantly higher than those of the control group (*P* < 0.05). Similarly, the results of the blood biochemical tests showed that the average level of TG in the case group was significantly higher (*P* < 0.05) than that in the control group.

**Table 3:** Physical examination of the participants and blood biochemical analysis

Variables	Case	Control	t/Z	*P*
	(n = 202)	(n = 216)		
BMI(kg/ m^2^)	26.35 ± 3.73	24.55 ± 3.28	− 3.589	<0.001*
SBP(mmHg)	140.74 ± 18.94	126.58 ± 13.65	−6.328	<0.001*
DBP(mmHg)	87.66 ± 11.91	79.87 ± 8.35	−5.829	<0.001*
TG(mM)	2.02 ± 1.08	1.93 ± 1.64	−2.391	0.017*
TC(mM)	4.82 ± 1.35	4.69 ± 1.17	−0.690	0.490
HDL-C(mmol/l)	1.03 ± 0.22	1.02 ± 0.24	−0.739	0.460
LDL-C(mmol/l)	2.95 ± 1.02	2.87 ± 0.81	−0.679	0.498
FPG(mmol/l)	8.00 ± 2.60	8.42 ± 2.43	−1.329	0.184

BMI = body mass index; SBP = systolic blood pressure; DBP = diastolic blood pressure; TG = triglyceride; TC = total cholesterol; HDL-C = high-density lipoprotein cholesterol; LDL-C = low-density lipoprotein; FPG = fasting plasma glucose. Significance: * = *P* < 0.05

### Hardy-Weinberg equilibrium

As shown in Table 4, the rs5182 (*P* = 0.839), rs5186 (*P* = 0.990) and rs35533650 (*P* = 0.987) the correct variation type were all consistent with Hardy-Weinberg equilibrium (*P* > 0.05) across the entire sample population.

**Table 4:** Hardy-Weinberg equilibrium status of the three *AT1R* gene the correct variation type

Genotype	Actual frequency	Theoretical frequency	χ^2^	*P*
rs5182			0.352	0.839
TT	239	236		
TC	151	156		
CC	28	26		
rs5186			0.020	0.990
AA	358	357		
AC	57	58		
CC	3	3		
rs35533650			0.026	0.987
AA	374	373		
AG	42	43		
GG	2	2		

### Genotypic and allele frequency of *AT1R* gene the correct variation type

As shown in Tables 5 and 6, the distribution of genotype (χ2 = 6.795, *P* = 0.033) and allele (χ2 = 6.555, *P* = 0.010) frequencies for the rs5182 polymorphism were significantly different between the case and control groups (*P* <0.05). Although the allele frequency (χ2 = 5.031, *P* = 0.025) of the T1878G polymorphism was significantly different between the two groups (*P* < 0.05), there was no significant difference in the genotype frequency (*P* > 0.05) for this polymorphism. In terms of the rs5186 polymorphism, there were no significant differences between the two groups with regard to genotype or allele frequency (*P* > 0.05).

**Table 5:** Genotypic frequency of *AT1R*

Genotype	Case	Control	χ^2^	*P*
	(*n* = 202)	(*n* = 216)		
rs5182			6.795	0.033*
TT	105(0.52)	134(0.62)		
TC	78(0.39)	73(0.34)		
CC	19(0.09)	9(0.04)		
rs5186			2.985	0.225
AA	177(0.88)	197(0.91)		
AC	23(0.11)	19(0.09)		
CC	2(0.01)	0(0)		
rs35533650			5.025	0.081
AA	165(0.82)	193(0.89)		
AG	35(0.17)	22(0.10)		
GG	2(0.01)	1(0.01)		

Significance: * = *P* < 0.05

**Table 6:** Analysis of the association between rs5182, rs5186 and rs35533650 and diabetes co-morbid with hypertension

Allele	Case	Control	P	OR (95%CI)
	(n = 202)	(n = 216)		
T573C				
TT	105(0.52)	134(0.62)		1
TC	78(0.39)	73(0.34)	0.301	1.323(0.778 ~ 2.251)
CC	19(0.09)	9(0.04)	0.042	3.219(1.042 ~ 9.941)
A1166C				
AA	177(0.88)	197(0.91)		1
AC	23(0.11)	19(0.09)	0.999	–
CC	2(0.01)	0(0)	0.186	1.860(0.741 ~ 4.669)
A1878G				
AA	165(0.82)	193(0.89)		1
AG	35(0.17)	22(0.10)	0.438	1.326(0.651 ~ 2.701)
GG	2(0.01)	1(0.01)	0.816	1.396(0.085 ~ 22.985)

Adjust sex, age, BMI, smoking and drinking.

### Association analysis between *AT1R* gene genotypes and diabetes co-morbid with hypertension

As shown in Table 6, after adjusting for sex, age, BMI, smoking, alcohol consumption and other variables by non-conditional logistic regression analysis, patients with the rs5182 polymorphism (CC genotype) had a significantly higher risk of diabetes mellitus with hypertension than did those with the TT genotype (OR = 3.219, 95% CI: 1.042 ~ 9.941, *P* = 0.042).

As shown in Table 6, after adjusting for sex, age, BMI, smoking alcohol consumption and other variables (such as confounding factors) in non-conditional logistic regression analysis, the rs5186 polymorphism of the *AT1R* gene was not associated with diabetes complicated with hypertension. There was no statistically significant difference in the risk of hypertension among patients with different A1166 genotypes. After adjusting for sex, age, BMI, smoking alcohol consumption and other variables, no association between the *AT1R* gene rs35533650 polymorphism and diabetes complicated with hypertension was observed by non-conditional logistic regression analysis (Table 6). Furthermore, there was no statistically significant difference in the risk of hypertension among patients with different A1878 genotypes.

### Association analysis between *AT1R* SNPs and systolic and diastolic blood pressure

Multiple linear regression was used to analyse associations of *AT1R* the correct variation type (rs5182, rs5186, rs35533650) with systolic blood pressure and diastolic pressure. In this analysis, systolic and diastolic blood pressure were used as dependent variables, whereas *AT1R* polymorphism sites, sex, age, BMI, smoking and drinking were used as independent variables (Tables 7, 8 and 9). After adjusting for sex, age, BMI, smoking, alcohol consumption, the rs5182 polymorphism of the *AT1R* gene was associated with diastolic pressure (*P* < 0.05) but not with systolic pressure (*P* > 0.05). Conversely, there was no correlation between *AT1R* gene rs5186 and rs35533650 the correct variation type and systolic or diastolic pressure (*P* > 0.05).

**Table 7:** Multivariate linear regression analysis of the rs5182 polymorphism with systolic pressure and diastolic pressure

Variables	SBP	DBP						
	β	S.E.	t	P	β	S.E.	t	P
rs5182	0.524	2.414	0.217	0.829	−0.232	0.107	−2.162	0.032*
Sex	−8.373	4.252	−1.969	0.051	−1.593	2.662	−0.598	0.551
Age	0.067	0.171	0.392	0.696	1.654	2.933	0.564	0.574
BMI	0.122	0.464	0.263	0.793	0.532	0.291	1.830	0.069
Smoking	−8.814	4.599	−1.917	0.057	−3.228	2.879	−1.121	0.264
Alcohol consumption	0.118	4.685	0.025	0.980	0.329	1.511	0.218	0.828

Significance: * = *P* < 0.05

**Table 8:** Multivariate linear regression analysis of the rs5186 polymorphism with systolic pressure and diastolic pressure

Variables	SBP	DBP						
	β	S.E.	t	P	β	S.E.	t	P
rs5186	−5.041	4.297	−1.173	0.243	−2.532	2.695	−0.940	0.349
Sex	−8.219	4.229	−1.944	0.054	−1.510	2.652	−0.570	0.570
Age	0.054	0.170	0.319	0.750	0.554	0.290	1.909	0.058
BMI	0.165	0.463	0.357	0.722	−0.238	0.107	−2.229	0.027*
Smoking	−9.500	4.576	−2.076	0.040*	−3.586	2.870	−1.250	0.214
Alcohol consumption	0.514	4.641	0.111	0.912	1.867	2.910	0.642	0.522

Significance: * = *P* < 0.05

**Table 9:** Multivariate linear regression analysis of the rs35533650 polymorphism with systolic pressure and diastolic pressure

Variables	SBP	DBP						
	β	S.E.	t	P	β	S.E.	t	P
rs35533650	0.524	2.414	0.217	0.829	1.000	2.367	0.422	0.673
Sex	−8.373	4.252	−1.969	0.051	−1.628	2.662	−0.612	0.542
Age	0.067	0.171	0.392	0.696	0.539	0.291	1.856	0.066
BMI	0.122	0.464	0.263	0.793	−0.227	0.108	−2.106	0.037*
Smoking	−8.814	4.599	−1.917	0.057	−3.291	2.860	−1.151	0.252
Alcohol consumption	0.118	4.685	0.025	0.980	1.662	2.918	0.640	0.570

Significance: * = *P* < 0.05

## Discussion

The role of genetic factors in the development and progression of diabetes mellitus complicated with hypertension has become an important research subject. The renin-angiotensin system plays an crucial role in water and electrolyte balance and in blood pressure regulation. Angiotensin II exerts its biological effects by binding to its receptor (ATR), the most important of which is the angiotensin II type 1 receptor (*AT1R*). The *AT1R* gene was isolated from the lymphocyte genomic library, and its coding region is contained in a single exon (no introns). *AT1R* encodes a protein of 359 amino acids that includes seven transmembrane functional regions. The *AT1R* amino acid sequence is highly conserved, and there is a high degree of identity between the *AT1R* gene sequence of humans with that of cattle and rats [20]. Several studies have suggested that the *AT1R* gene is closely related to diabetes and hypertension [21, 22]. However, the genetic basis for this association is still not well understood, and the search for the correct variation type within the *AT1R* gene, which might be highly associated with diabetes complicated with hypertension, is ongoing.

Bonnardeaux et al. [8] focused on the correct variation type found in the coding region and the 3′ untranslated region of the *AT1R* gene. In the present study, sixty patients with a family history of hypertension were analysed for the presence of single-strand conformation the correct variation type. Five polymorphic sites were identified (T573 → C, A1062 → G, A1166 → C, G1517 → T, A1878 → G), of which the rs5186 polymorphism is the most studied. Although research has found a link between the presence of the rs5186 polymorphism and the occurrence of hypertension [23–25], the correlation between this polymorphism and diabetes complicated with hypertension remains controversial. For example, Leasage et al. [12] did not find an association between the rs5186 polymorphism and diabetes complicated with hypertension. These results are in agreement with our results, which indicate no statistically significant difference in the presence of *AT1R* rs5186 between the case and control groups (*P* > 0.05). Unconditional logistic regression analysis also revealed no association between the A1166 polymorphic site and diabetes mellitus with hypertension, nor was it associated with systolic or diastolic blood pressure. Nonetheless, Xue Yaoming et al. [26] have suggested a correlation between *AT1R* rs5186 and diabetes complicated with hypertension and elevated systolic blood pressure. Moreover, Karpov et al. [27] suggested that the rs5186 polymorphism contributes to hypertension in patients with diabetes. Our studies contradict the findings of these two studies [26, 27]. The possible reasons for the inconsistency are different target regions, lifestyle choices, population genetics and sample sizes, which may affect genotypic and allelic frequencies and ultimately influence linkage disequilibrium. For example, all participants in the present study were native Han inhabitants of Inner Mongolia.

The relationship between the *AT1R* gene rs5182 polymorphism and diabetes mellitus with hypertension has not been studied to the same extent as has the rs5186 polymorphism. Regardless, the results of the present study suggest that the rs5182 genotype frequency (χ2 = 6.795, *P* = 0.033) and allele frequency (χ2 = 6.555, *P* = 0.010) differ between patients with diabetes mellitus complicated with hypertension and diabetic patients without hypertension (*P* < 0.05). Furthermore, non-conditional logistic regression analysis suggested 3.219-fold increased risk of diabetes mellitus complicated by hypertension with the rs5182 polymorphism (CC genotype) (OR = 3.219, 95% CI: 1.042–9.941, *P* = 0.042) compared with T573T (TT genotype) (*P* < 0.05). At the same time, multivariate linear regression analysis showed that the *AT1R* gene rs5182 polymorphism was associated with diastolic blood pressure (*P* < 0.05). These results suggest that *AT1R* rs5182 is associated with diabetes complicated with hypertension in the Han population of Inner Mongolia.

The involvement of the rs35533650 polymorphism of the *AT1R* gene in the pathogenesis of diabetes complicated with hypertension is also under investigation, and our results suggested that the allele frequency of rs35533650 (χ2 = 5.031, *P* = 0.025) in diabetic patients with hypertension was significantly different from that in diabetic patients without hypertension (*P* < 0.05). However, subsequent non-conditional logistic regression analysis did not reveal an association between the rs35533650 polymorphism in patients with diabetes with hypertension. The possible reason for this inconsistency might be the level of complexity of both diabetes and hypertension, whereby susceptibility genes have only small incremental contributions to a phenotype. In the present study, we did not find an association between the rs35533650 polymorphism and diabetes complicated with hypertension.

National and international studies to date report that the main risk factor for diabetes complicated with hypertension is being overweight or obese. The results of this study suggested that the average BMI in the case group was significantly higher (BMI = 26.35 ± 3.73) kg / m^2^ than that in the control group (BMI = 24.55 ± 3.28) kg / m^2^) (*P*<0.05). Our logistic regression analysis results also indicated that BMI is a risk factor for patients with diabetes complicated with hypertension. Colditz et al. analysed the BMI of 11,361 U.S. women aged 30–55 years [28] and found that women with a BMI between 23 and 23.9 kg/m2 had a 3.6-fold higher risk of developing diabetes after 8 years than did those with a BMI < 22 kg/m^2^. Additionally, the same study found that weight gain after 18 years was also a risk factor for diabetes: a weight gain of 20 to 35 kg increased the risk by 11.3 times, and a weight gain of more than 35 kg increased the risk by 17.3 times. According to another survey, the prevalence of hypertension in both men and women increases with BMI: when BMI < 25 kg / m^2^, the prevalence of hypertension in men and women was 15%, but when BMI ≥ 30 kg / m^2^, the prevalence of hypertension in both men and women rose to 42 and 38%, respectively [29]. In general, overweight and obesity are important risk factors for cardiovascular and cerebrovascular diseases [30]. Backer et al. showed that weight gain was related to the incidence and mortality of cardiovascular and cerebrovascular diseases; thus, it has been suggested that weight loss in those with obesity might reduce the risk of cardiovascular and cerebrovascular diseases [31]. A cohort study of male workers in the Shougang region, China, estimated a risk-adjusted odds ratio of 1.06 (95% confidence interval: 1.02–1.09) for each unit of increase in BMI [32]. In addition, the study by Song et al., which spans 11 cohorts from four European countries, showed that the mortality caused by cardiovascular and cerebrovascular diseases increased with greater BMI. The same study notes that when male and female BMI values are at the same level, the corresponding cardiovascular and cerebrovascular disease-related mortality is higher in males than in females [33].

## Conclusions

In conclusion, the results of this study suggest that the rs5182 polymorphism of the *AT1R* gene is associated with an increased risk of diabetes complicated by hypertension. Diabetic patients with a CC genotype at this site have a higher risk of developing hypertension than do those with a TT genotype. The rs5186 and rs35533650 the correct variation type do not appear to be associated with an increased risk of diabetes complicated with hypertension.

## Supplementary information

**Additional file 1.** The questionnaire of diabetes with hypertension and diabetes.

## Data Availability

All data generated or analysed during this study are included in this published article [and its supplementary information files].

## Abbreviations

*AT1R*: angiotensin II type 1 receptor
T2DM: type 2 diabetes mellitus
BMI: body mass index
SBP: systolic blood pressure
DBP: diastolic blood pressure
TG: triglyceride
TC: total cholesterol
HDL-C: high-density lipoprotein cholesterol
LDL-C: low-density lipoprotein cholesterol
FPG: fasting plasma glucose
WHO: World Health Organization
RAS: renin-angiotensin system
AGT: angiotensinogen
REN: renin
ACE: angiotensin-converting enzyme
HWE: Hardy-Weinberg equilibrium
OR: odds ratio
CI: confidence interval
